# SPP1+macrophages promote fibroblast-to-myofibroblast transformation during hypoxia in deep fascia of acute compartment syndrome

**DOI:** 10.3389/fimmu.2025.1588926

**Published:** 2025-10-02

**Authors:** Liujie Zheng, Guoqiang Li, Jingcheng Cao, Zihang Zhao, Haofei Wang, Qi Dong, Zhiyong Hou

**Affiliations:** ^1^ Department of Orthopedic Surgery, Hebei Medical University Third Hospital, Shijiazhuang, Hebei, China; ^2^ Engineering Research Center of Orthopedic Minimally Invasive Intelligent Equipment, China Ministry of Education, Shijiazhuang, China; ^3^ Key Laboratory of Biomechanics of Hebei Province, Shijiazhuang, Hebei, China; ^4^ National Health Commission (NHC) Key Laboratory of Intelligent Orthopedic Equipment, Shijiazhuang, Hebei, China; ^5^ Key Laboratory of Precise Assessment, Diagnosis, and Treatment of Soft Tissue Injury of Hebei Province, Shijiazhuang, China

**Keywords:** acute compartment syndrome, hypoxia, fascia, macrophage, fibroblast

## Abstract

**Background:**

Acute compartment syndrome (ACS) is a life-threatening condition characterized by elevated intracompartmental pressure leading to ischemia, hypoxia and tissue necrosis. We have observed an increase in SPP1+macrophages and fibroblast activation in the deep fascia of ACS patients. However, the mechanisms underlying the pathological changes in terms of macrophage activation and fibroblast responses remain poorly understood.

**Objectives:**

This study aims to investigate whether hypoxia induces SPP1^+^ macrophages in the deep fascia of ACS, and to elucidate how these macrophages contribute to fibroblast activation and myofibroblast transformation.

**Methods:**

Macrophages were cultured under normoxic and hypoxic conditions, with or without SPP1 siRNA transfection. The concentration of SPP1 in the macrophage-conditioned media was determined using an ELISA assay. The culture supernatant from each condition was then applied to fibroblasts, which were subsequently analyzed for mRNA and protein expression of fibroblast activation markers by qRT-PCR and WB, respectively.

**Results:**

Hypoxia significantly upregulated SPP1 expression in macrophages, as well as in the macrophage-conditioned media. Moreover, hypoxia-stimulated macrophages promoted fibroblast expression of ACTA2, CTGF, collagen I, collagen III, and FN1 at both mRNA and protein levels. This effect was reversed by SPP1 siRNA transfection.

**Conclusions:**

Hypoxia induces SPP1+macrophages, which in turn activate fibroblasts, driving myofibroblast transformation in ACS. Targeting this pathway may provide a potential therapeutic strategy for mitigating fibrosis and improving outcomes in ACS.

## 1 Introduction

Acute compartment syndrome (ACS) is a severe and life-threatening condition caused by elevated intracompartmental pressure, which leads to ischemia, hypoxia and tissue necrosis (1). The deep fascia, a vital connective tissue structure surrounding muscles, blood vessels, and nerves, plays a crucial role in the pathophysiology of ACS (2). However, the mechanisms driving pathological alterations in the deep fascia during ACS, particularly those involving macrophage activation and fibroblast responses, remain poorly understood.

In our previous study, we found a significant accumulation of SPP1+ macrophages accompanied by fibroblast activation in the deep fascia of ACS patients compared to healthy individuals (3). SPP1 (Secreted Phosphoprotein 1), also known as osteopontin, is a secreted glycoprotein widely expressed in a variety of cell types that plays a momentous role in inflammation, tissue repair and fibrosis (4). It regulates cell adhesion, migration, and extracellular matrix remodeling through interactions with receptors such as integrins, the hyaluronan receptor, and CD47 (5). Macrophages are among primary sources of SPP1, especially during inflammatory responses, where it contributes to the regulation of inflammation, tissue repair, and fibrosis (6, 7). Fibroblasts are the main cell type of the deep fascia and are central to its structure and pathophysiology. They are responsible for synthesizing collagen, elastin, glycosaminoglycans and other matrix proteins that provide structural support, thereby playing a crucial role in maintaining, repairing and remodeling its extracellular matrix (ECM) of deep fascia (8, 9).

The hypoxic microenvironment within the affected compartment is a hallmark of ACS, resulting from compromised blood flow (10). Hypoxia is a potent regulator of cellular responses, particularly in immune cells such as macrophages. Recent single-cell profiling studies have identified SPP1+ macrophages in the hypoxic regions of various diseases, including hepatocellular carcinoma, lung squamous cell carcinoma, and recurrent miscarriage (11–13). Additionally, hypoxia-ischemia has been reported to upregulate SPP1 expression in Iba-1+/TMEM119+ microglia and Iba-1+/TMEM119- monocytes/macrophages (14). These findings suggest that hypoxia may similarly enhance SPP1 expression in macrophages within the deep fascia of ACS patients. Furthermore, studies have demonstrated that platelet-instructed SPP1+ macrophages drive myofibroblast activation, playing a significant role in chronic kidney fibrosis (15). These findings suggest that SPP1-expressing macrophages may also contribute to fibroblast activation in ACS. We therefore hypothesize that the hypoxic microenvironment in the deep fascia of ACS patients induces macrophages to upregulate SPP1 expression, which in turn activates fibroblasts through paracrine signaling, thereby promoting tissue remodeling and fibrosis.

This interplay between hypoxia, SPP1-expressing macrophages, and fibroblast activation may represent a critical pathway in the development of ACS. A better understanding of how hypoxia induces SPP1 production in macrophages and how this process influences fibroblast behavior provide a new insights into the molecular mechanisms underlying tissue damage and repair in ACS. Ultimately, this knowledge may facilitate the development of novel diagnostic markers and therapeutic strategies aimed at targeting macrophage–fibroblast crosstalk to prevent or mitigate ACS-related complications.

## 2 Materials and methods

### 2.1 Reagents

α-SMA (USA, AF1032, diluted 1:1000) antibody, collagen I (USA, AF7001, diluted 1:1000) antibody, fibronectin (USA, AF5335, diluted 1:500) antibody, CTGF (USA, DF7091, diluted 1:500) antibody and collagen III (USA, AF5457, diluted 1:500) antibody were purchased from Affinity. PMA (USA, HY-18739) was purchased from MCE. Lipo8000™ Transfection Reagent (China, C0533) was purchased from Biyuntian (Shanghai Biyuntian Biotechnology Co., LTD., Shanghai, China). The osteopontin (OPN/SPP1) ELISA kit (China, RX105857H) was purchased from Ruixinbio.

### 2.2 Cell culture

THP-1 cells were cultured in RPMI-1640 (+10%FBS; +1% Penicillin/Streptomycin; 2 mM L-Glutamine), and treated with 100 nM PMA for 48h to induce macrophage-like adhesion. Cells were then incubated in PMA-free medium for 24h to obtain resting macrophages, at 37 °C with 5% CO_2_ and 95% humidity. SPP1 siRNA transfection was conducted with Lipo8000™ Transfection Reagent following the instructions. Human dermal fibroblast cells (CP-H103, Wuhan Punosai Life Technology Co., LTD, China) were cultured in DMEM+ FBS 15% +P/S 1% at 37 °C with 5% CO_2_ and 95% humidity. Macrophages were divided into four groups and cultured for 24h: the control group (normoxic, 21% O_2_), the hypoxia group (hypoxic, 5% O_2_), the si-SPP1 group (transfected with SPP1 siRNA and then cultured under hypoxic conditions, 5% O_2_), and the negative control group (transfected with scrambled siRNA and then cultured under hypoxic conditions, 5% O_2_). After 24h, the culture supernatant was collected for subsequent detection and use. The macrophage supernatant from each group was then added to the fibroblast culture medium corresponding to the four conditions (control, hypoxia, si-SPP1, and negative control). After 24h of incubation, fibroblasts were collected for further analysis.

### 2.3 Measurement of SPP1 levels by ELISA

Macrophages were cultured under normoxic and hypoxic conditions, with or without SPP1 siRNA transfection. The concentration of SPP1 in the macrophage-conditioned media was quantified using a human osteopontin (OPN/SPP1) ELISA kit according to the manufacturer’s instructions.

### 2.4 Quantitative real-time polymerase chain reaction

Total cellular RNA was extracted using the Trizol reagent (SB-MR009, ShareBio, China). Reverse transcription was then performed using the All-in-One First-Strand Synthesis MasterMix (with dsDNase) kit (EG15133S, iScience, China) to synthesize cDNA. The resulting cDNA was subsequently amplified with Taq SYBR Green qPCR Premix (Universal) (EG20117M, iScience, China) under the following cycling conditions: initial polymerase activation at 95 °C for 30 s, followed by 40 cycles of 95 °C for 15 s and 60 °C for 30 s. Gene expression levels were normalized to β-actin mRNA. The sequences of the primers used are listed in **Table 1**.

**Table 1:** Primers designed for quantitative real-time PCR.

Gene	Primer	Sequence(5’-3’)
Hu-β-actin(96)	Forward	CCCTGGAGAAGAGCTACGAG
	Reverse	GGAAGGAAGGCTGGAAGAGT
Hu-collagen I(137)	Forward	CACCAATCACCTGCGTACAG
	Reverse	GCAGTTCTTGGTCTCGTCAC
Hu-collagen III(101)	Forward	TCCACTCCATAACGCTCCTC
	Reverse	GTGGCCTTGGTATGTGCTTT
Hu-CTGF(182)	Forward	TTAGCGTGCTCACTGACCTG
	Reverse	GCCACAAGCTGTCCAGTCTA
Hu-FN1(158)	Forward	GGTACAGGGTGACCTACTCG
	Reverse	GGGCTGGCTCTCCATATCAT
Hu-ACTA2(85)	Forward	GGAATCCTGTGAAGCAGCTC
	Reverse	CTTACAGAGCCCAGAGCCAT

### 2.5 Western blot

Macrophages and fibroblasts were washed three times with PBS and lysed using RIPA lysis buffer (Biosharp, BL504A, China). Proteins were separated by sodium dodecyl sulfate–polyacrylamide gel electrophoresis (SDS-PAGE) and then transferred onto a PVDF membrane (Millipore, IPVH00010, United States). Protein bands were visualized using the ECL Chemiluminescent Substrate Reagent Kit (GK10008, GLPBIO, China), and signal detection was performed with JS-M6P imaging system (Peiqing Technology Co., Ltd., Shanghai, China). Band intensity was quantified using ImageJ software (version 1.52).

### 2.6 Statistical analysis

All analyses were conducted using SPSS software (version 24.0). One-way ANOVA was used to assess significant differences in mRNA and protein expression among treatment groups, followed by Bonferroni’s *post-hoc* test. Data are presented as mean ± standard deviation (SD). A p-value of < 0.05 was considered statistically significant.

## 3 Results

### 3.1 Hypoxia upregulates macrophage expression of SPP1

**Figure 1:**
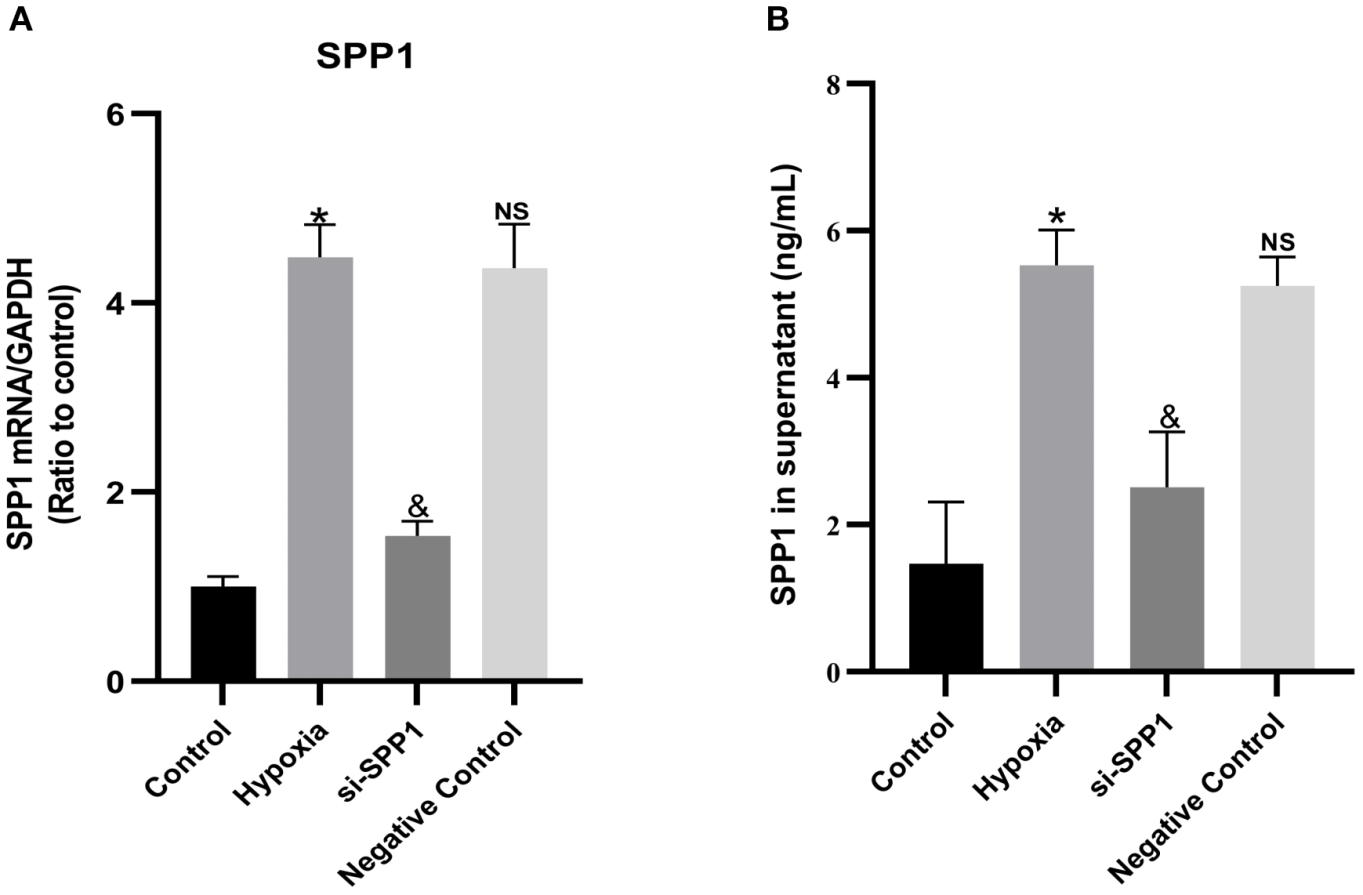
Hypoxia upregulates macrophage expression and secretion of SPP1. Macrophages were divided into four groups and cultured for 24 hours: the control group (normoxic, 21% O_2_), the hypoxia group (hypoxic, 5% O_2_), the si-SPP1 group (transfected with SPP1 siRNA before being cultured under hypoxic conditions, 5% O_2_), and the negative control group (transfected with scrambled siRNA before being cultured under hypoxic conditions, 5% O_2_). SPP1 gene expression in macrophages was assessed by qRT-PCR **(A)**, and SPP1 protein concentration in the macrophage-conditioned supernatant was measured by ELISA **(B)**. Data are expressed with mean ± SD; *p < 0.05: Hypoxia group *vs* Control group, &p < 0.05: si-SPP1 group *vs* Hypoxia group, ns, Negative control group *vs* Hypoxia group. All experiments were repeated three times independently.

As shown in **Figures 1A, B**, the mRNA expression of SPP1 was significantly increased compared to the control group, However, transfection of macrophages with SPP1 siRNA suppressed Hypoxia-induced SPP1 mRNA expression in macrophages and (p < 0.05).

### 3.2 Inhibition of SPP1 suppressed macrophage induced fibroblast mRNA expression of ACTA2, CTGF, collagen I, collagen III and FN1

**Figure 2:**
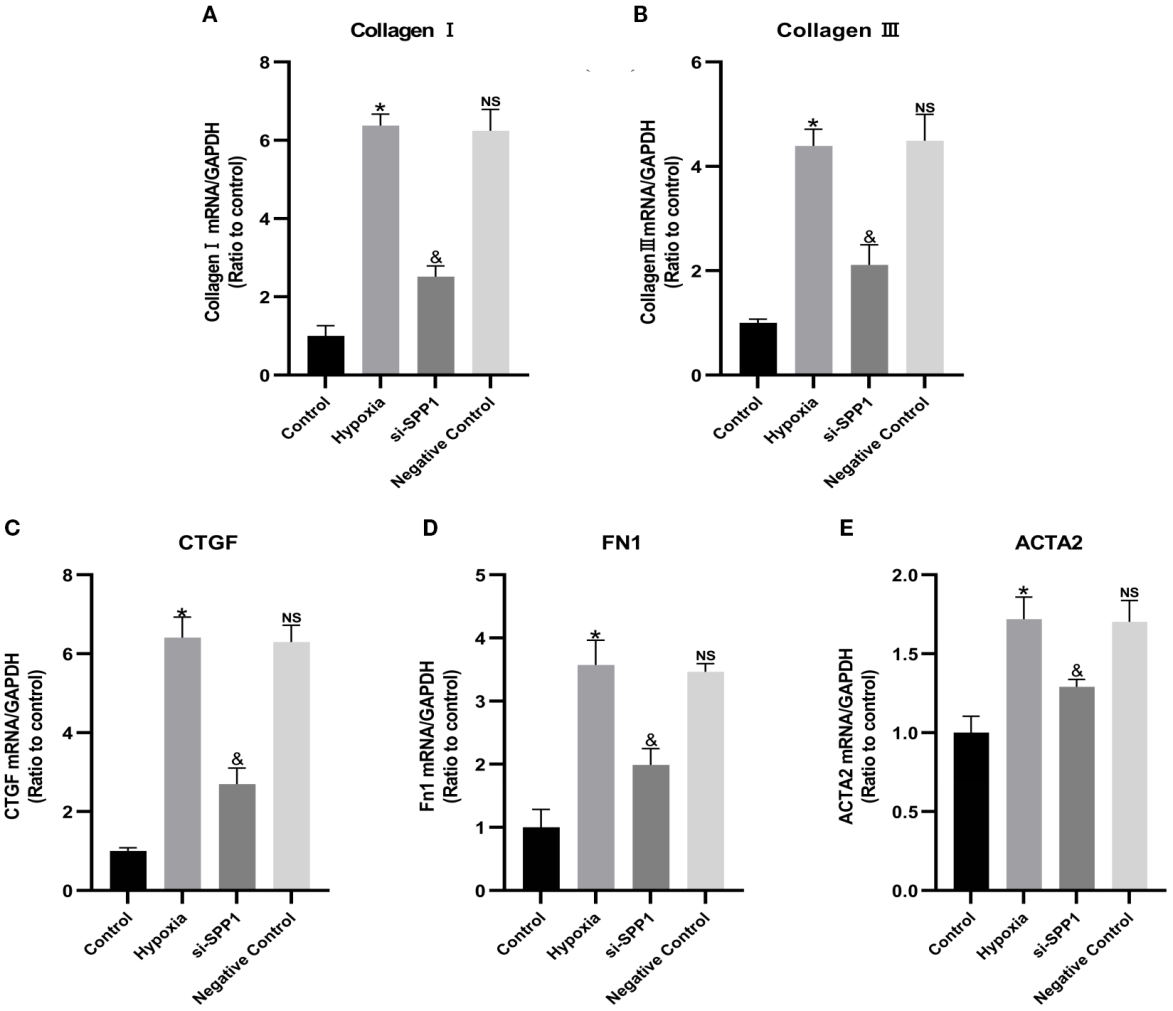
Inhibition of SPP1 attenuates macrophage-induced fibroblast mRNA expression of ACTA2, CTGF, collagen I, collagen III and FN1. Macrophage-conditioned supernatant from each group was added to fibroblast culture medium under the four conditions (control, hypoxia, si-SPP1 and negative control) for 24 hours. Subsequently, the mRNA expression levels of ACTA2, CTGF, collagen I, collagen III and FN1 in fibroblasts were then analyzed by qRT-PCR **(A–E)**. Data are expressed with mean ± SD; **(A–E)**, *p < 0.05: Hypoxia group *vs* Control group, ^&^p < 0.05: si-SPP1 group *vs* Hypoxia group, ns, Negative control group *vs* Hypoxia group. All experiments were repeated three times independently.

As shown in **Figures 2A–E**, hypoxia-stimulated macrophages significantly increased the mRNA expression of ACTA2, CTGF, collagen I, collagen III, and FN1 in fibroblasts. However, transfection of macrophages with SPP1 siRNA significantly reduced the hypoxia-induced expression of these genes (p < 0.05).

### 3.3 Inhibition of SPP1 suppressed macrophage induced fibroblast protein expression of α-SMA, CTGF, collagen I, collagen III and FN

**Figure 3:**
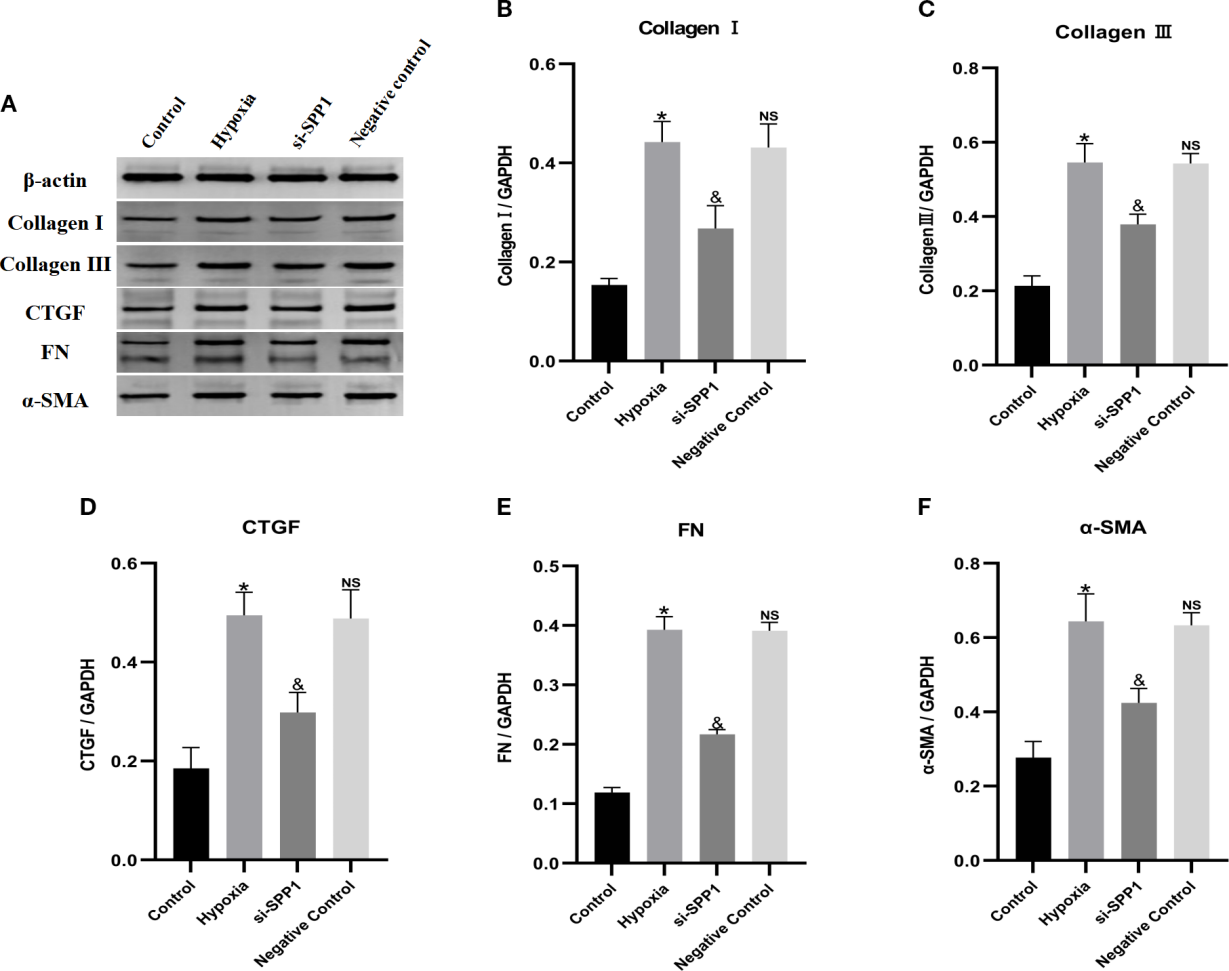
Inhibition of SPP1 suppresses macrophage-induced fibroblast protein expression of α-SMA, CTGF, collagen I, collagen III and FN. Macrophage-conditioned supernatant from each group was added to the fibroblast culture medium under the four conditions (control, hypoxia, si-SPP1 and negative control) for 24 hours. The protein expression levels of α-SMA, CTGF, collagen I, collagen III and FN in fibroblasts were analyzed by Western blot **(A–F)**. Data are expressed with mean ± SD; **(B–F)**, *p < 0.05: Hypoxia group *vs* Control group, ^&^p < 0.05: si-SPP1 group *vs* Hypoxia group, ns, Negative control group *vs* Hypoxia group. All experiments were repeated three times independently.

As shown in **Figures 3A–F**), hypoxia-stimulated macrophages significantly increased the protein expression of α-SMA, CTGF, collagen I, collagen III, and FN in fibroblasts. In contrast, transfection of macrophages with SPP1 siRNA significantly attenuated the hypoxia-induced upregulation of these proteins (p < 0.05).

## 4 Discussion

Acute compartment syndrome (ACS) is a severe and potentially limb-threatening condition characterized by increased intracompartmental pressure within a closed muscle compartment, leading to ischemia, hypoxia, and tissue damage. The deep fascia, a critical structure in ACS, is actively involved in tissue repair and fibrosis, with macrophages and fibroblasts playing central roles. Our previous single-cell profiling found an accumulation of SPP1+ macrophages and activated fibroblasts in the deep fascia of ACS patients, suggesting their potential involvement in ACS pathogenesis. In the present study, we investigated the role of SPP1+ macrophages in the deep fascia of ACS, focusing on their impact on fibroblast activation under hypoxic conditions. We found that hypoxia, a hallmark of ACS, significantly upregulated SPP1 expression in macrophages. These hypoxia-induced macrophages, in turn, promoted fibroblast activation and tissue remodeling, as evidenced by increased expression of fibrosis-related genes and proteins, including ACTA2, CTGF, collagen I, collagen III, and FN1.

Hypoxia, a hallmark of ACS, is a potent inducer of cellular responses, particularly in immune cells. The hypoxic microenvironment has been reported to activate macrophages, which play a key role in tissue remodeling and repair (16, 17). Our findings show that hypoxia increases SPP1 expression in macrophages, which was consistent with previous studies (13). It has been reported that elevated levels of HIF1α in macrophages can upregulate SPP1 expression (18, 19). So it was possible that that hypoxia induces HIF1α activation, which in turn leads to increased SPP1 expression in macrophages. Previous studies have highlighted that SPP1 can promote fibroblast activation by regulating key fibrotic markers such as α-SMA, collagen production, and fibronectin synthesis (20, 21). Our findings further support the role of SPP1+ macrophages in mediating the fibrotic response in ACS could through paracrine signaling, emphasizing the crucial role of the macrophage-fibroblast interaction in the pathophysiology of ACS. Furthermore, elevated SPP1 expression in fibroblasts has been shown to drive myofibroblast differentiation via the PI3K/Akt/mTOR and TGF-β signaling pathways in pulmonary and renal fibrosis, respectively (22–24). However, the precise mechanisms by which SPP1 induces fibroblast-to-myofibroblast transformation through paracrine signaling remain unclear and warrant further investigation. Importantly, we found that inhibition of SPP1 expression in macrophages by siRNA significantly attenuated fibroblast activation and fibrosis-related gene and protein expression. These findings suggest that SPP1 is a critical mediator of macrophage-induced fibroblast activation and myofibroblast differentiation. Targeting the SPP1 pathway may therefore represent a promising therapeutic approach to mitigate fibrosis in ACS, improve tissue recovery, and reduce long-term complications.

However, this study has several limitations. First, although we found that hypoxia-induced SPP1+ macrophages drive fibroblast activation, the specific molecular pathways by which hypoxia regulates SPP1 expression in macrophages, such as HIFs, remain unclear and need further exploration. In addition, the exact mechanisms through which SPP1 activates fibroblasts and promotes their differentiation into myofibroblasts are not fully understood. Second, the hypoxic conditions (5% O_2_) used in our study may not completely replicate the oxygen gradients present in ACS tissues, and future research should consider more clinically relevant models. Finally, while we focused on SPP1-mediated signaling between macrophages and fibroblasts, possible feedback loops between these cells and the contribution of other extracellular matrix components also merit further exploration.

In conclusion, SPP1+ macrophages play a pivotal role in driving fibroblast activation and fibrosis in ACS. Future studies should further elucidate the molecular mechanisms underlying macrophage–fibroblast interactions to advance our understanding of ACS pathophysiology and to facilitate the development of more effective treatments.

## Data Availability

The data analyzed in this study is subject to the following licenses/restrictions: The data that support the findings of this study are available from the corresponding author upon reasonable request. Requests to access these datasets should be directed to LZ 390582959@qq.com.

## References

[1] Guo J Yin Y Jin L Zhang R Hou Z Zhang Y. Acute compartment syndrome: Cause, diagnosis, and new viewpoint. Med (Baltimore). (2019) 98:e16260. doi: 10.1097/MD.0000000000016260, PMID: 31277147 PMC6635163

[2] Wang H Liu Y Xu S Wang T Chen X Jia H. Proteomics analysis of deep fascia in acute compartment syndrome. PLoS One. (2024) 19:e0305275. doi: 10.1371/journal.pone.0305275, PMID: 38950026 PMC11216580

[3] Wang T Long Y Ma L Dong Q Li Y Guo J. Single-cell RNA-seq reveals cellular heterogeneity from deep fascia in patients with acute compartment syndrome. Front Immunol. (2023) 13:1062479. doi: 10.3389/fimmu.2022.1062479, PMID: 36741388 PMC9889980

[4] King EM Zhao Y Moore CM Steinhart B Anderson KC Vestal B. Gpnmb and Spp1 mark a conserved macrophage injury response masking fibrosis-specific programming in the lung. JCI Insight. (2024) 9:e182700. doi: 10.1172/jci.insight.182700, PMID: 39509324 PMC11665561

[5] Wu G Li X Seo H McLendon BA Kramer AC Bazer FW. Osteopontin (OPN)/secreted phosphoprotein 1 (SPP1) binds integrins to activate transport of ions across the porcine placenta. Front Biosci (Landmark Ed). (2022) 27:117. doi: 10.31083/j.fbl2704117, PMID: 35468676

[6] Qi J Sun H Zhang Y Wang Z Xun Z Li Z. Single-cell and spatial analysis reveal interaction of FAP+ fibroblasts and SPP1+ macrophages in colorectal cancer. Nat Commun. (2022) 13:1742. doi: 10.1038/s41467-022-29366-6, PMID: 35365629 PMC8976074

[7] Sathe A Mason K Grimes SM Zhou Z Lau BT Bai X. Colorectal cancer metastases in the liver establish immunosuppressive spatial networking between tumor-associated SPP1+ Macrophages and fibroblasts. Clin Cancer Res. (2023) 29:244–60. doi: 10.1158/1078-0432.CCR-22-2041, PMID: 36239989 PMC9811165

[8] Pirri C Caroccia B Angelini A Petrelli L Piazza M Biz C. Evidence of renin-angiotensin system receptors in deep fascia: A role in extracellular matrix remodeling and fibrogenesis? Biomedicines. (2022) 10:2608. doi: 10.3390/biomedicines10102608, PMID: 36289870 PMC9599236

[9] Plikus MV Wang X Sinha S Forte E Thompson SM Herzog EL. Fibroblasts: Origins, definitions, and functions in health and disease. Cell. (2021) 184:3852–72. doi: 10.1016/j.cell.2021.06.024, PMID: 34297930 PMC8566693

[10] Witthauer L Cascales JP Roussakis E Li X Goss A Chen Y. Portable oxygen-sensing device for the improved assessment of compartment syndrome and other hypoxia-related conditions. ACS Sens. (2021) 6:43–53. doi: 10.1021/acssensors.0c01686, PMID: 33325684

[11] Bao S Chen Z Qin D Xu H Deng X Zhang R. Single-cell profiling reveals mechanisms of uncontrolled inflammation and glycolysis in decidual stromal cell subtypes in recurrent miscarriage. Hum Reprod. (2023) 38:57–74. doi: 10.1093/humrep/deac240, PMID: 36355621

[12] Fan G Xie T Li L Tang L Han X Shi Y. Single-cell and spatial analyses revealed the co-location of cancer stem cells and SPP1+ macrophage in hypoxic region that determines the poor prognosis in hepatocellular carcinoma. NPJ Precis Oncol. (2024) 8:75. doi: 10.1038/s41698-024-00564-3, PMID: 38521868 PMC10960828

[13] Hao B Dong H Xiong R Song C Xu C Li N. Identification of SLC2A1 as a predictive biomarker for survival and response to immunotherapy in lung squamous cell carcinoma. Comput Biol Med. (2024) 171:108183. doi: 10.1016/j.compbiomed.2024.108183, PMID: 38422959

[14] Xin D Li T Chu X Ke H Liu D Wang Z. MSCs-extracellular vesicles attenuated neuroinflammation, synapse damage and microglial phagocytosis after hypoxia-ischemia injury by preventing osteopontin expression. Pharmacol Res. (2021) 164:105322. doi: 10.1016/j.phrs.2020.105322, PMID: 33279596

[15] Hoeft K Schaefer GJL Kim H Schumacher D Bleckwehl T Long Q. Platelet-instructed SPP1+ macrophages drive myofibroblast activation in fibrosis in a CXCL4-dependent manner. Cell Rep. (2023) 42:112131. doi: 10.1016/j.celrep.2023.112131, PMID: 36807143 PMC9992450

[16] Wang Y Zhang Y Li J Li C Zhao R Shen C. Hypoxia induces M2 macrophages to express VSIG4 and mediate cardiac fibrosis after myocardial infarction. Theranostics. (2023) 13:2192–209. doi: 10.7150/thno.78736, PMID: 37153746 PMC10157727

[17] Kang Y Xu L Dong J Yuan X Ye J Fan Y. Programmed microalgae-gel promotes chronic wound healing in diabetes. Nat Commun. (2024) 15:1042. doi: 10.1038/s41467-024-45101-9, PMID: 38310127 PMC10838327

[18] Luo H She X Zhang Y Xie B Zhang S Li Q. PLIN2 promotes lipid accumulation in ascites-associated macrophages and ovarian cancer progression by HIF1α/SPP1 signaling. Adv Sci (Weinh). (2025) 12(12):e2411314. doi: 10.1002/advs.202411314"10.1002/advs.202411314, PMID: 39921309 PMC11948008

[19] Jie H Wang B Zhang J Wang X Song X Yang F. Uncovering SPP1+ Macrophage, neutrophils and their related diagnostic biomarkers in intracranial aneurysm and subarachnoid hemorrhage. J Inflammation Res. (2024) 17:8569–87. doi: 10.2147/JIR.S493828, PMID: 39539729 PMC11559423

[20] Fei C Chen Y Tan R Yang X Wu G Li C. Single-cell multi-omics analysis identifies SPP1+ macrophages as key drivers of ferroptosis-mediated fibrosis in ligamentum flavum hypertrophy. biomark Res. (2025) 13:33. doi: 10.1186/s40364-025-00746-6, PMID: 40001138 PMC11863437

[21] Huang R Hao C Wang D Zhao Q Li C Wang C. SPP1 derived from silica-exposed macrophage exosomes triggers fibroblast transdifferentiation. Toxicol Appl Pharmacol. (2021) 422:115559. doi: 10.1016/j.taap.2021.115559, PMID: 33961903

[22] Yue B Xiong D Chen J Yang X Zhao J Shao J. SPP1 induces idiopathic pulmonary fibrosis and NSCLC progression via the PI3K/Akt/mTOR pathway. Respir Res. (2024) 25:362. doi: 10.1186/s12931-024-02989-7, PMID: 39369217 PMC11456247

[23] Ding H Xu Z Lu Y Yuan Q Li J Sun Q. Kidney fibrosis molecular mechanisms Spp1 influences fibroblast activity through transforming growth factor beta smad signaling. iScience. (2024) 27:109839. doi: 10.1016/j.isci.2024.109839, PMID: 39323737 PMC11422156

[24] Li H Li P Shen Q Zhu Z Yang M Zhang X. Nfil3 contributes to renal fibrosis by activating fibroblasts through directly promoting the expression of Spp1. Biochim Biophys Acta Mol Basis Dis. (2025) 1871(4):167741. doi: 10.1016/j.bbadis.2025.167741"10.1016/j.bbadis.2025.167741, PMID: 39986442

